# Veterinary Medical Association of New York County

**Published:** 1894-02

**Authors:** John E. Ryder

**Affiliations:** Secretary


### VETERINARY MEDICAL ASSOCIATION OF NEW YORK COUNTY.

A meeting was called to order on January 25th, 1894. by Dr. Thos. Giffen, County Secretary of the New York State Veterinary Medical Society, who in a short address stated that the meeting was called to organize an association in the county of New York, upon the following basis, viz.:

1. To hold regular meetings to discuss professional subjects for mutual improvement. and to improve the status of the veterinary profession by bringing its members into more intimate relationship, and consolidating them as a distinctive professional body in the community.

2. To regulate veterinary affairs and methods in the education and practice of veterinary medicine to be in harmony with and conformity to the regulations of the New York State Veterinary Society and the U. S. Veterinary Medical Association.

He then announced that Dr. W. H. Hoskins, of Philadelphia, Pa., President of the U. S Veterinary Medical Association, was present, and moved that he be made temporary chairman, which was unanimously carried.

*Dr. Hoskins*, on taking the chair, congratulated the gentlemen present on the step they were about to take, pointing out the benefits that were to be derived by it individually and collectively; then, after speaking briefly of other societies, announced the first business in order would be the election of officers.

*Dr. O'Shea* nominated for President Dr. R. S. Huidekoper.

*Dr. Cattanach, Sr.*, nominated for Vice-President Dr. J. L. Robertson.

*Dr. Neher* nominated for Secretary Dr. J. E. Ryder.

The above named officers were elected by acclamation.

For Treasurer three nominations were made, consisting of Drs. Giffen, Loomes and Farley. The chair appointed Drs. Hanson and Neher as tellers, and a ballot was taken; twenty-eight votes were cast, of which Dr. Giffen received 24, Dr. Loomes 3, Dr. Farley 1. The chair thereupon declared Dr. Giffen elected Treasurer, which upon a motion duly seconded was made unanimous.

Moved by Dr. Turner and seconded by Dr. Loomes that a committee of nine be appointed by the chair to draft a constitution and by-laws for this organization. Carried.

The chair appointed Dr. J. L. Robertson, Chairman, Drs. O'Shea, J. S. Cattanach, Neher, Sherwood, Turner, Farley, Gill and Becket.

Moved by C. C. Cattanach, and seconded by Dr. Giffen, that a recess of fifteen minutes be taken for the committee to select name for the organization, officers, when and where to meet, dues, etc. Carried. After the recess the committee reported as follows:

That the name of the organization be The Veterinary Medical Association of New York County.

That the officers be President, Vice-President, Secretary and Treasurer, and a committee of five members who shall be known as the Board of Censors, to be appointed by the President at each annual meeting.

Meetings to be held on the first Tuesday in each month, except during July, August and September.

The meeting to be held in December to be known as the Annual Meeting, at which time all officers are to be elected for the ensuing year, to take office at the following meeting.

Members to consist of

1. Charter Members: All those practitioners of Veterinary Medicine who assist in the organization of the above Association, and conform to the regulations formulated by a two-thirds vote of those in attendance at the meeting of organization.

2. Future Members: To be elected members, who shall be graduates of Veterinary Institutions which are legally entitled to grant Veterinary diplomas, and which shall be recognized by this Association as properly equipped, qualified and conducted for the same.

Dues: Each member of the Association shall pay an initiation fee of $5, and yearly dues of $2, payable annually in advance.

On hearing the report an amendment was offered by Dr. Giffen that registered men who are non-graduates shall be eligible for membership providing their application was received within two months, after which members will have to be qualified, registered and graduated men.

After considerable discussion by several members the amendment was seconded and carried.

Report of committee with above amendment was received and approved.

Moved and seconded that the Committee on By-Laws be continued, and that they report more completely at next meeting. Carried.

Moved by Dr. O'Shea, that a committee of five be appointed by the Chair to confer with those members of the profession having in contemplation the formation of another society. Motion seconded and carried.

Moved and seconded, that Dr. W. H. Hoskins be elected an honorary member of this Association. Unanimously carried. A vote of thanks was also unanimously extended to him.

The Chair appointed the following members as Censors for 1894: Dr. Liautard, chairman, Drs. O'Shea, Gill, Loomes and Sherwood.

Following as Committee on Conference: Dr. J. L. Robertson, chairman, Drs. Ferster, Giffen, Swan and Neher.

The following constitute the charter members of the Association:

| | | |
|---|---|---|
| Drs. Birdsall, | Drs. Burget, | Drs. Burchstead, |
| R. Buckley, | P. Burns, | C. C. Cattanach, |
| J. J. Cattanach, | W. J. Coates, | W. A. Conklin, |
| T. Delaney, | R. H. Dickson, | T. H. Doyle, |
| J. H. Ferster, | O. C. Farley, | S. S. Field, |
| T. Giffen, | H. D. Gill, | S. K. Johnson, |
| R. S. Huidekoper, | W. H. Jackson, | J. C. Leighton, |
| E. Loomes, | A. F. Liautard, | R. W. Hickman, |
| H. Neher, | T. Ogle, | A. O'Shea, |
| C. A. Parkerson, | E. A. Parsons, | J. L. Robertson, |
| J. E. Ryder, | P. Richards, | T. Sherwood, |
| Wm. Swan, | A. Sherk, | C. Sielman, |
| J. B. Thompson, | T. S. Cattanach. | |

All of whom were present except the following: Drs. Birdsall, Burget, Burns, Coates, Field, Johnson, Jackson, Leighton, Liautard, Ogle, Parsons, Sherk.

No further business, meeting adjourned to meet Tuesday, Feb. 6th, '94.

JOHN E. RYDER, *Secretary.*

